# ADRC-Based Control Method for the Vascular Intervention Master–Slave Surgical Robotic System

**DOI:** 10.3390/mi12121439

**Published:** 2021-11-25

**Authors:** Wei Zhou, Shuxiang Guo, Jin Guo, Fanxu Meng, Zhengyang Chen

**Affiliations:** 1 School of Life Science, Beijing Institute of Technology, Beijing 100081, China; zhouwei@bit.edu.cn (W.Z.); mengfanxu@bit.edu.cn (F.M.); chenzhengyang@bit.edu.cn (Z.C.); 2 Key Laboratory of Convergence Medical Engineering System and Healthcare Technology, Ministry of Industry and Information Technology, School of Life Science, Beijing Institute of Technology, Beijing 100081, China; 3 Faculty of Engineering, Kagawa University, 2217-20 Hayashi-cho, Takamatsu 760-8521, Japan

**Keywords:** active disturbance rejection control (ADRC), ADRC-based control method, vascular interventional surgery, master–slave robotic system, surgical safety

## Abstract

In vascular interventional surgery, surgeons operate guidewires and catheters to diagnose and treat patients with the assistance of the digital subtraction angiography (DSA). Therefore, the surgeon will be exposed to X-rays for extended periods. To protect the surgeon, the development of a robot-assisted surgical system is of great significance. The displacement tracking accuracy is the most important issue to be considered in the development of the system. In this study, the active disturbance rejection control (ADRC) method is applied to guarantee displacement tracking accuracy. First, the core contents of the proportional–integral–derivative (PID) and ADRC methods are analyzed. Second, comparative evaluation experiments for incremental PID and ADRC methods are presented. The results show that the ADRC method has better performance of than that of the incremental PID method. Finally, the calibration experiments for the ADRC control method are implemented using the master–slave robotic system. These experiments demonstrate that the maximum tracking error is 0.87 mm using the ADRC method, effectively guaranteeing surgical safety.

## 1. Introduction

Cardiovascular and cerebrovascular diseases are characterized by a high disability rate and high mortality, and are considered the leading killers of human health according to the World Health Statistics 2020 reported by the World Health Organization. At present, vascular interventional surgery is used clinically to diagnose and treat cardiovascular and cerebrovascular lesions, such as angiography and stent placement. Although the surgeons wear lead clothes weighing 20 kg to treat the patients during the clinical operation, the surgeons still suffer from X-ray radiation when performing digital subtraction angiography (DSA). Some studies have shown that if surgeons are exposed to X-rays for a long time, they are prone to suffer from diseases such as cancerous tumors, eye diseases (lens opacity), and bone diseases [1,2,3]. Robot-assisted surgery is a promising method to avoid X-ray radiation and its associated complications. Moreover, robot-assisted surgery can reduce the surgical failure rate caused by the surgeon’s hand tremor. In robot-assisted surgery, high displacement tracking accuracy is the most important issue in ensuring operating safety. Therefore, a study of the control method is necessary to improve the tracking accuracy.

The multifunctional vascular intervention surgical robotic system (VISRS) is a promising way to assist surgeons in operation [4]. A number of advanced VISRSs have been developed and commercialized, such as the CorPath^®^ Robot System (Corindus Robotics Inc., Waltham, MA, USA) [5], Sensei Robotic System (Hansen Medical Inc., Mountain View, CA, USA) [6], Amigo Robot System (Catheter Precision Inc., Ledgewood, NJ, USA) [7], and Niobe (Stereotaxis Inc., St. Louis, MO, USA) [8]. In addition, Hansen Medical was acquired by Auris, and their technology portfolio was absorbed into the Monarch endovascular robot (now belonging to Auris) [9]. Although all these systems can operate the guidewire and catheter, they cannot provide tactile perception. Therefore, there are some safety risks.

In addition to these commercial systems, there are also studies that deal with the above-mentioned issues. Shi et al. developed a VISRS with a haptic perception interface based on a spring-force generator to operate the catheter [10]. However, the circumferential force was not considered in this system. Jin et al. developed a tactile sensing robot-assisted system using a master manipulator with force feedback [11]. However, the force feedback was generated by magnetorheological fluid controlled by a magnetic field generator, which is not convenient. The steerable catheter and the haptic function in [12] can reduce the operating time and guarantee surgical safety. Bao et al. proposed a slave manipulator [13,14]. This manipulator achieved the collaboration of the catheter and guidewire-driven device. Moreover, the performance of this manipulator was calibrated using animal and clinical trials. However, the master manipulator used in the system does not mimic the surgeon’s operating habits during the clinical operation, which increases the time required to learn a new device. Hao et al. developed a remote-controlled robotic system with multiple functional slave robots that can inject contrast agents but cannot insert the guidewire and catheter [15].

Moreover, there have been some studies on safety strategies. Omisore et al. proposed a robotic catheter system to access the human cardiac area through the radial vasculature [16]. Additionally, this system can characterize backlash behavior and eliminate it. Zhang et al. proposed collision detection with haptic cues shown on a PC screen in the developed catheter-guidewire operating system, which can help the surgeon to complete the surgery safely and successfully [17]. Guo et al. proposed a VISRS with force-visual feedback, which can provide two-dimensional information to ensure intraoperative security [18,19]. In addition, other methods have been implemented to improve the performance of VISRS. Kang et al. proposed a hydraulically steerable guidewire with a diameter of 400 μm. In addition, this guidewire consists of a flexible eccentric tube to realize two different curvatures [20]. Pancaldi et al. proposed a navigation strategy using microengineered endovascular probes driven by fluid energy. This method is promising for accessing deep brain regions through an endovascular path [21]. Zhou et al. analyzed the natural behavior of interventionalists during a conventional percutaneous coronary intervention to promote the future design of human–robot interfaces [22]. Guo et al. designed a contactless catheter-sensing method that simultaneously senses translational and rotational motions using a passive marker with four feature point groups [23]. Zhao et al. used a deep-learning algorithm to realize the navigation and perception of the robot and the surgeon’s operational skills assessment [24,25,26,27].

However, only a few studies have looked at the control method in the VISRS. Hu proposed a control strategy to improve the performance of robot-assisted cardiovascular surgery [28]. In this method, generalized predictive control (GPC) is used to suppress the effects of time-varying delay and parameter identification error. Moreover, a terminal sliding mode controller was designed to improve the robustness of the system under consideration. Jin et al. proposed a system using a proportional–integral–derivative (PID) control method [29,30]. Yang et al. adopted the fuzzy PID control method to improve the tracking accuracy; however, the maximal error of the system is close to 2 mm, which is dangerous for patients [31]. Wang et al. proposed a VISRS using the PID control algorithm [32]. The master manipulator was a joystick, which did not conform to the surgeon’s operating habits. Haidegger et al. used the force-based control algorithm using surgical robotics [33]. This method can provide a higher quality human–machine interaction, more realistic sensory feedback and telepresence. Haidegger et al. proposed a stochastic approach to improve the precision of integrated setups [34]. This method was helpful to increase the safety and reliability of all procedures, ease the surgeon’s task and potentially reduce operating time. Jayender et al. proposed a force-based control algorithm to control the insertion of the catheter. This method can reduce exposure to harmful X-ray radiation [35]. Guo et al. proposed a robust control algorithm to reduce the displacement error. In addition, the method had good performance through the stability analysis using the lyapunov function method [36].

According to the current research status, the main research contents consist of the development of the master manipulator and the slave manipulator, the method of navigation methods, and the warning strategies for safe operation. Moreover, research on the control method makes it possible to improve the tracking accuracy. However, the tracking accuracy of some studies using the PID control algorithm was not ideal. Therefore, it is necessary to study a control method to improve the control performance of the robot-assisted surgical system.

In the up-to-date studies on the control method in the VISRS, the PID controller was the main control method applied in this field. In addition, PID control methods with few parameters have been widely used in industrial control applications. However, PID control method has some limitations, such as: error computation, noise degradation in the derivative control, oversimplification, and the loss of performance in the control law [37]. Moreover, the ADRC method is a promising method to overcome the weakness of the PID method. Peng et al. combined the ADRC with the consensus algorithm to control multi-degree-of-freedom (DOF) parallel electrical manipulator systems, demonstrating better performance [38]. Łakomy et al. improved the performance of the ADRC by using a new observer structure to address the problem caused by high-frequency measurement noise in practice [39]. Chi et al. proposed an improved ADRC, using local dynamic linearization to realize the nonlinear system affine to control the input in the globally Lipschitz nonlinear discrete-time system [40]. Wang et al. presented a back propagation (BP) neural network-based ADRC to address these problems, such as strong couplings and nonlinear and unstable disturbances. This control method can achieve decoupling control in a three-degree-of-freedom six-pole active magnetic bearing [41]. Lu et al. proposed a load-adaptive two-loop drive system based on an improved position–speed integrated ADRC with a parameter fuzzy self-tuning method. In addition, this method can realize a high-speed and high-precision position servo system for a permanent magnet synchronous motor [42]. Sun et al. proposed a quantitative tuning rule for the time-delayed ADRC structure based on the typical first-order plus time-delay model, which shows better closed-loop tracking performance [43]. By analyzing the results of the studies, all these related studies show that the ADRC method can improve the displacement accuracy. Therefore, it is promising to apply the ADRC method to the VISRS to realize high-quality master–slave tracking accuracy and operating stability.

The contribution of this study is that it is the first implementation of the ADRC control method in the VISRS. The performance of the ADRC method is better than that of the PID method. Moreover, the ADRC method can achieve a shorter adjustment time and high tracking accuracy. The remainder of this paper is organized as follows: The master–slave VISRS is demonstrated in Section 2. In addition, the displacement measurement assembly of the master manipulator is evaluated. In Section 3, the principles of PID and ADRC are introduced. Moreover, comparative experiments of these two methods are implemented. In Section 4, the performance of the VISRS using the ADRC method is evaluated. In Section 5, the discussion and conclusions of this study are presented.

## 2. Master–Slave VISRS Description

### 2.1. Principle of the VISRS

**Figure 1:**
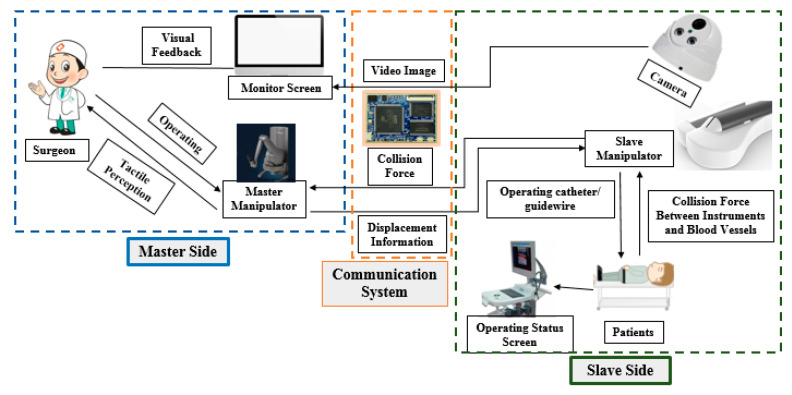
Overview of the master–slave VISRS.

The master–slave VISRS consists of three parts: master side, slave side, and communication system, as shown in Figure 1. The master and slave sides are placed in two different rooms. On the master side, the surgeon operates the master manipulator in a safe space without radiation. The operation is assisted by video images on the monitor screen. Moreover, the displacement information generated by the master manipulator is used to control the slave manipulator on the slave side through the communication system. On the slave side, the slave manipulator operates the catheter guidewire to diagnose and treat the patients during surgery. Simultaneously, the camera is used to monitor the intraoperative state on the slave side. In addition, the collision force information and video image generated by the camera are transmitted to the master side through the communication system. Moreover, the communication system is a connector between the master and slave sides. Both haptic perception and visual feedback are necessary to guarantee surgical safety in the VISRS. The above information can assist the surgeon in locating the position and recognizing the intraoperative state of the guidewire/catheter. However, the most important factor influencing intraoperative safety is the tracking accuracy, which refers to the displacement accuracy when the slave manipulator follows the master manipulator.

### 2.2. Description of the Master Manipulator

**Figure 2:**
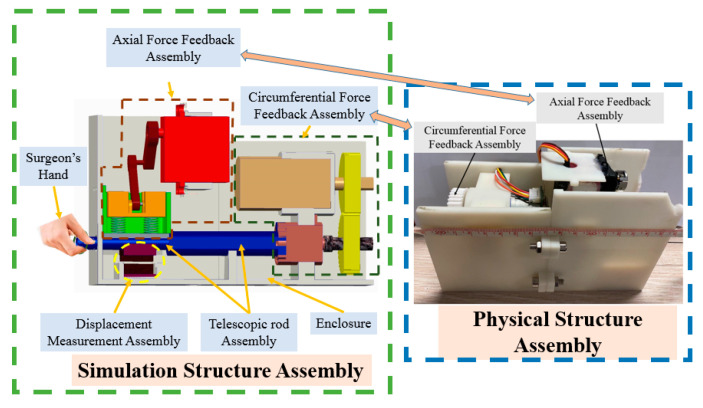
Structure of the master manipulator.

**Figure 3:**
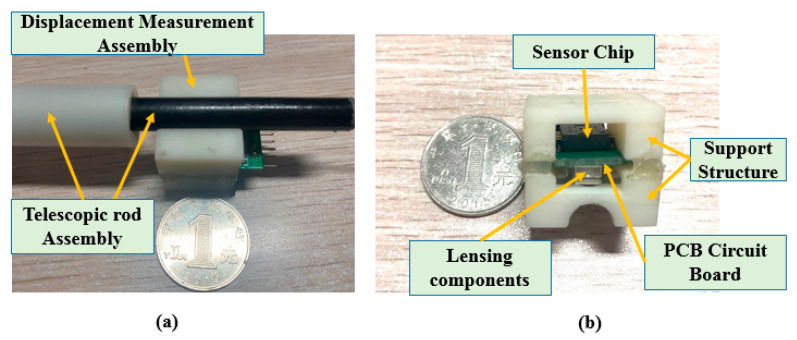
Structure of the DMS. (**a**) Assembly relationship between the DMS and telescopic rod assembly. (**b**) Details of the physical structure of the DMS.

The master manipulator developed in this study is shown in Figure 2. The simulation and physical structures are also presented. The advantage of this master manipulator is that it is consistent with the operating habits of traditional clinical surgery. The operating habits is that the surgeon operates the guidewire/catheter through three actions (push, retraction, and rotation along the axial direction) from the femoral artery to the target position in traditional vascular interventional surgery [29]. The surgeon uses the thumb, index finger, and middle finger to hold the telescopic rod. Moreover, with the movement of the fingers, the telescopic rod is pulled, retracted, or rotated along the axial direction. In addition, the displacement information was measured simultaneously. This master manipulator consists of an axial force feedback assembly, a circumferential force feedback assembly, a displacement measurement assembly (DMS), a telescopic rod assembly, and an enclosure. In this study, to improve the tracking accuracy is the main focus of this study. The tracking accuracy is related to the DMS. Therefore, we evaluate the performance of DMS in this study, while the performance of the axial and the circumferential force feedback assemblies will be evaluated in a future work. The core component of the DMS is an optical mouse sensor (PAW3515DB-TJZA, PixArt Imaging Inc., Taiwan, China). The working principle of the optical mouse sensor is presented in [44,45]. The physical structure of the DMS is shown in Figure 3. This DMS consists of a PCB circuit board, sensor chip, lensing components, and a support structure. In addition, the PCB circuit board was designed and manufactured based on the function of the sensor chip.

### 2.3. Description of the Slave Manipulator

**Figure 4:**
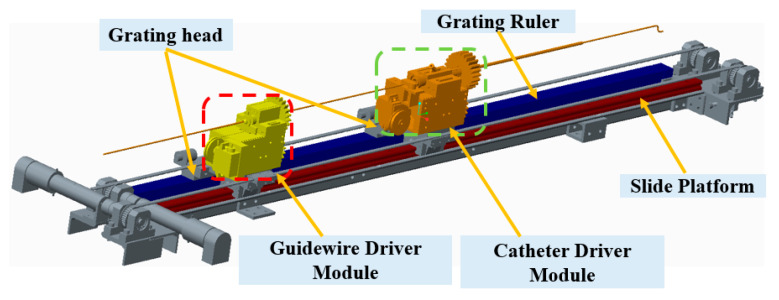
Structure of the slave manipulator.

The slave manipulator shown in Figure 4 includes the slide platform, guidewire driver module, and catheter driver module [13,14,46]. This manipulator was evaluated in animal and clinical experiments. The guidewire driver module is used to clamp the guidewire and drive the rotation and insertion movement of the guidewire. The functionality of the catheter driver module is similar to that of the guidewire driver module, which is used for the catheter. A slide platform with a grating ruler was used to guarantee the coaxiality of the guidewire and catheter controllers. In addition, a grating ruler is used to record real-time displacement. In particular, this slave manipulator can realize the cooperation of the guidewire and catheter driver module to satisfy the requirements of surgery.

### 2.4. Calibration Experiments for the DMS of the Master Manipulator

**Figure 5:**
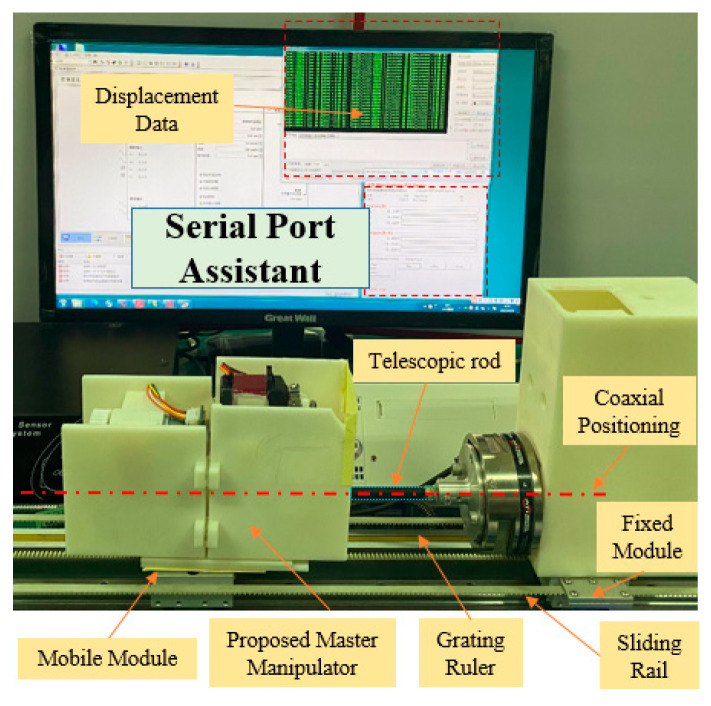
Calibration experimental platform setup.

**Figure 6:**
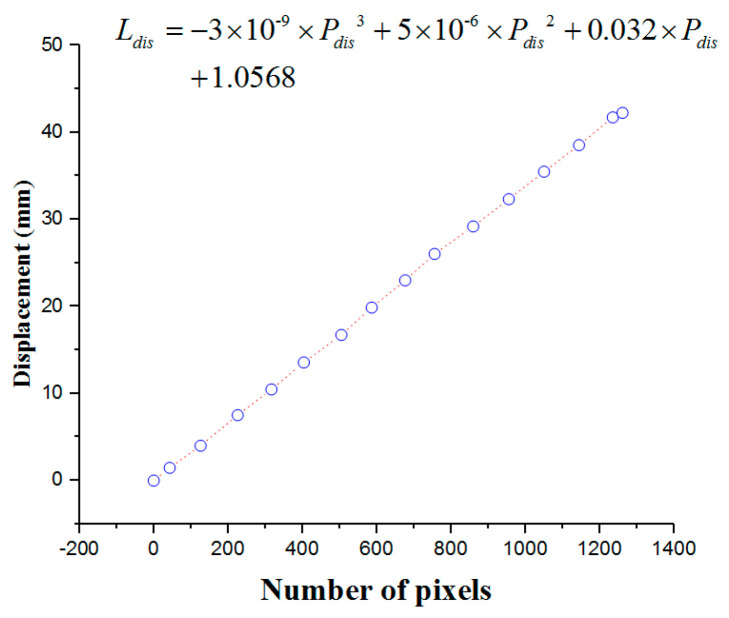
Calibration results for the DMS.

In this section, calibration experiments are implemented to build the relationship between the number of pixels and displacement. The experimental platform is shown in Figure 5, consisting of the mobile module, fixed module, and serial port assistant. The proposed master manipulator is mounted on the mobile module, which was driven by a DC motor (525506, Maxon Motor, Switzerland). The mobile and fixed modules are mounted on the same sliding rail to guarantee coaxiality. Coaxiality is very important in guaranteeing measurement accuracy. A grating ruler is used to measure the real-time displacement of the mobile module. In the calibration experiments, the mobile module is driven to achieve a uniform motion. Simultaneously, the telescopic rod is pushed along the sliding rail. The displacements and number of pixels are recorded through the serial port assistant. The calibration results are presented in Figure 6.

The relationship between the displacement and number of pixels is shown in Equation (1):

(1) $$L_{dis}=-3\times 10^{-9}\times P_{dis}{}^{3}+5\times 10^{-6}\times P_{dis}{}^{2}+0.032\times P_{dis}+1.0568$$

where $L_{dis}$ is the displacement and $P_{dis}$ is the number of pixels.

## 3. Working Principles of the Control Method

The PID control method is a very popular and effective method in automatic control fields. It has only three parameters to be modified to obtain a better operational state. The output of the incremental PID control method is the increment of the controlled variable, which can avoid the accumulative error that influences the control accuracy. After determining the proportional, integral, and differential coefficients, the output is calculated based on the three consecutive errors. In addition, the principle of the incremental PID control method is presented [47]. However, the adaptive ability of the incremental PID control method is not excellent; that is, the parameters should be modified if the working environment changes. In vascular interventional surgery, there are large differences in the endovascular environments, comprising blood flow speed and vascular flexibility of different patients. Moreover, the ADRC method is promising for addressing the aforementioned issues. In this section, the working principles of ADRC are first demonstrated in detail. Comparative experiments of the two control methods are implemented.

### 3.1. Description of the ADRC Method

**Figure 7:**
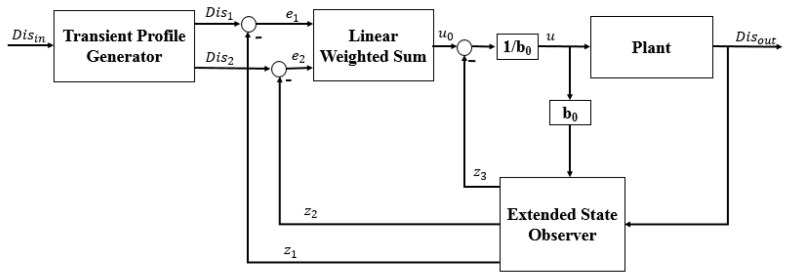
Structure of the ADRC method.

The ADRC method does not depend on a specific model of a controlled plant [37]. Moreover, the ADRC can provide a smooth transition from the current position to the target position. The structure of the ADRC (as shown in Figure 7) consists of four parts: the transient profile generator, extended state observer, linear error feedback controller, and compensation controller. The transient profile generator is used to provide a smooth operation process. The extended state observer is used to extract the system status and disturbance information. The linear error feedback controller is used to provide the error control variable. The compensation controller compensates for the disturbance. The complete algorithm of the ADRC is as follows [37]:

(1) The transient profile generator is depicted by Equation (2):
  (2) $$\{\begin{array}{l} e=Dis_{1}-Dis_{in}\\ fh=fhan\left(e,Dis_{2},r_{0},h\right)\\ Dis_{1}=Dis_{1}+hDis_{2}\\ Dis_{2}=Dis_{2}+hfh \end{array}$$
  where $Dis_{in}$ is the reference value, $Dis_{1}$ is the transient value, $Dis_{2}$ is the differential value of $Dis_{1}$, fh is the maximum speed synthesis function, $r_{0}$ is the speed factor, and h is the filter factor. The $r_{0}$ term is used to control the tracking speed, and h can filter the jitter. The output value is $Dis_{out}$.
(2) The extended state observer is demonstrated by Equation (3):
  (3) $$\{\begin{array}{l} e=z_{1}-Dis_{out},fe=fal\left(e,\alpha_{1},\delta \right),fe_{1}=fal\left(e,\alpha_{2},\delta \right),\\ z_{1}=z_{1}+h\left(z_{2}-\beta_{01}e\right),\\ z_{2}=z_{2}+h\left(z_{3}-\beta_{02}fe+b_{0}u\right),\\ z_{3}=z_{3}+h\left(-\beta_{03}fe_{1}\right), \end{array}$$
  where $z_{1}$, $z_{2}$ and $z_{3}$ are the status observer values of the $Dis_{1}$, $Dis_{2}$ and the total disturbance of the system (including the internal and external disturbances), respectively. The $\beta_{01}$, $\beta_{02}$ and $\beta_{03}$ are the scale parameters, which are calculated using Equation (4). The $\omega_{0}$ term is the bandwidth of the state observer. $b_{0}$ is the compensating parameter. The value of fal(e,α,δ) is calculated using Equation (5):
  (4) $$\{\begin{array}{c} \beta_{01}=3\omega_{0}\\ \beta_{02}=3\omega_{0}{}^{2}\\ \beta_{03}=\omega_{0}{}^{3} \end{array}$$
  (5) $$fal\left(e,\alpha ,\delta \right)=\{\begin{array}{cc} \frac{e}{\delta^{1-\alpha }} & \left|e\right|\leq \delta \\ {\left|e\right|}^{\alpha }sign\left(e\right), & \left|e\right|>\delta \end{array}$$
(3) The state error feedback law is given by Equation (6):
  (6) $$\{\begin{array}{l} e_{1}=Dis_{1}-z_{1},e_{2}=Dis_{2}-z_{2}\\ u_{0}=k_{1}\times e_{1}+k_{2}\times e_{2} \end{array}$$
  where $u_{0}$ is the plant of error feedback controlled, and $k_{1}$ and $k_{2}$ are the coefficients of the error and error derivatives, respectively.
(4) The disturbance compensation process is calculated by Equation (7):
  (7) $$u=u_{0}-\frac{z_{3}\left(t\right)}{b_{0}}$$

### 3.2. Comparative Experiments

**Figure 8:**
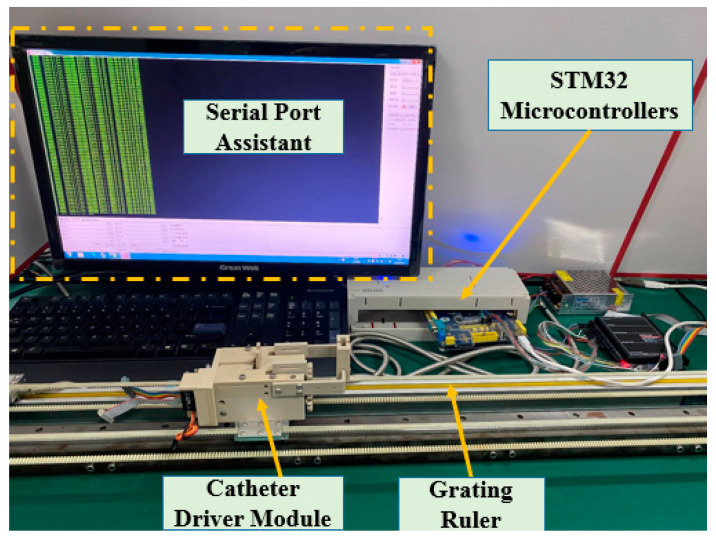
Experimental setup for the comparative experiments.

**Figure 9:**
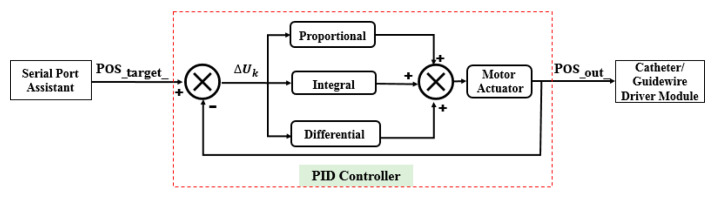
Structure of the PID controller used in the robot-assisted system.

**Figure 10:**
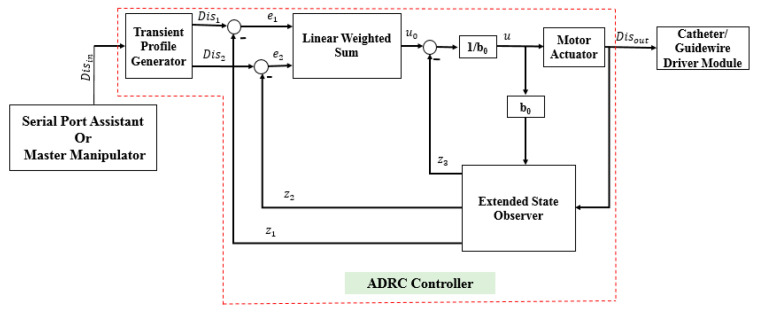
Structure of the ADRC controller used in the robot-assisted system.

**Figure 11:**
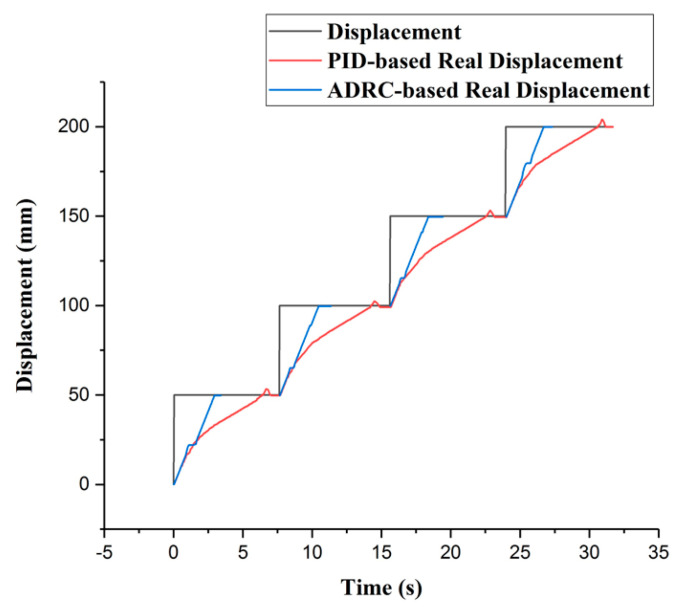
Comparative experimental results.

In this section, some experiments are implemented to compare the performance of the incremental PID and the ADRC control methods. The experimental setup is illustrated in Figure 8. The structures of the PID and ADRC controllers are shown in Figure 9 and Figure 10, respectively. The P, I, and D of the PID control method are tuned to 22,100,000, 210,000, and 1000, respectively. According to linear ADRC controller tuning method in [48], $\omega_{0}$, $k_{1}$, $k_{2}$ and $b_{0}$ are 0.01, 160, 20, and 50, respectively. In the PID controller, the ${\mathrm{Pos}}_{\_target\_}$, $∆U_{k}$, and ${\mathrm{Pos}}_{\_out\_}$ refer to the target displacement, error, and output displacement, respectively. In addition, $Dis_{in}$, u, and $Dis_{out}$ in the ADRC controller refer to the given target displacement, duty ratio of the pulse-width modulation (PWM), and output displacement, respectively. The motor is a linear actuator. In these experiments, the grating ruler is used to record the real displacements of the catheter driver module. A serial port assistant is used to set the target displacement through the STM32 microcontrollers. In addition, the overshoot, modifying time, and accuracy were considered as the evaluation indicators. During the experiments, the target position values were set up by a serial port assistant. The target position values include 50 mm, 100 mm, 150 mm, and 200 mm, which refer to the movement displacements of the guidewire/catheter-driven module. The experimental results are shown in Figure 11.

**Figure 12:**
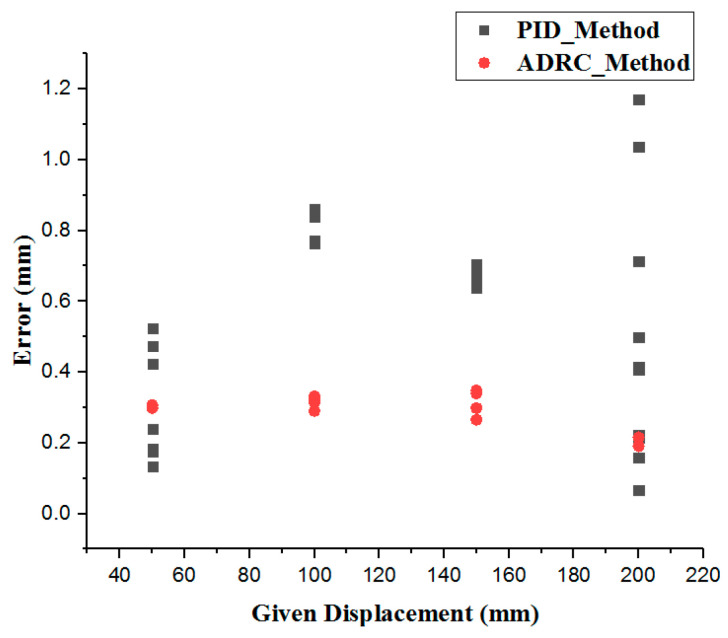
Error distributions of the ADRC and the incremental PID methods.

Figure 11 shows that the maximum modifying time is 7.82 s and the minimum modifying time is 7.39 s, using the PID method. In addition, using the ADRC method, the maximum modifying time is 3.24 s and the minimum modifying time is 3.00 s. Therefore, the ADRC method is better than the PID method in response speed. In Figure 12, the errors are calculated by Equation (8):

(8) $$Dis_{error}=\left|Dis_{target}-Dis_{real}\right|$$

where $Dis_{error}$ is the absolute value of the displacement error, $Dis_{target}$ is the target displacement given by the serial port assistants, and $Dis_{real}$ is the real displacement measured by the grating ruler. The maximum error is 1.20 mm using the PID method and 0.55 mm using ADRC method. The minimum error is 0.05 mm using the PID method and 0.18 mm using ADRC method. Here, error refers to steady-state error.

Some overshoot phenomena are observed when using the PID method. The maximum overshoot is more than 4.00 mm, which is very dangerous in robot-assisted surgery [31]. Moreover, there are phenomena in which the real displacement becomes stuck in the middle of the transient using the ADRC control method. This phenomenon is caused by the observed fluctuations in the value of the state observer. The nonuniform dynamic friction of the slide platform and the lag phenomenon of the observation state can cause fluctuations. In summary, the accuracy and response speed of the ADRC are better than those of the PID method. Moreover, the ADRC does not have an overshoot, which shows good stability. The reason for these properties is that the extended state observer in the ADRC method can compensate for the controlled plant of the linear error feedback controller. In addition, in robot-assisted surgery, different patients have different intravascular environments. The friction of the vasculature is also different. Moreover, abrasion of the slave manipulator is likely to occur. Because of the disturbances caused by these two reasons, the PID method should modify the coefficients. In contrast, the ADRC parameters are universal [37]. Therefore, the ADRC control method is more promising for guaranteeing operating safety, especially in VISRS.

## 4. Experiments and Results

**Figure 13:**
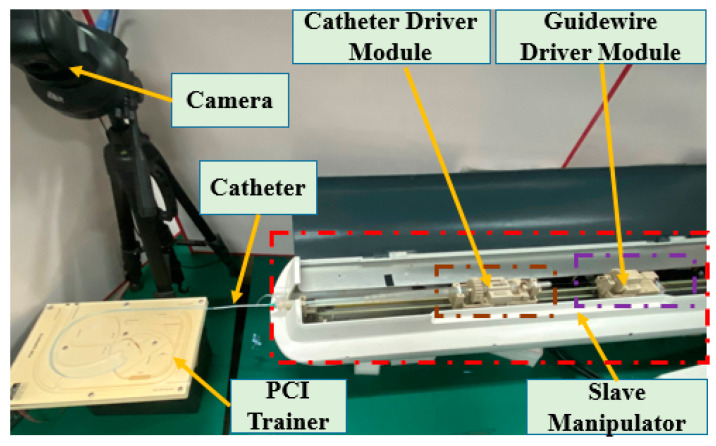
Experimental setup for the evaluation experiments.

**Figure 14:**
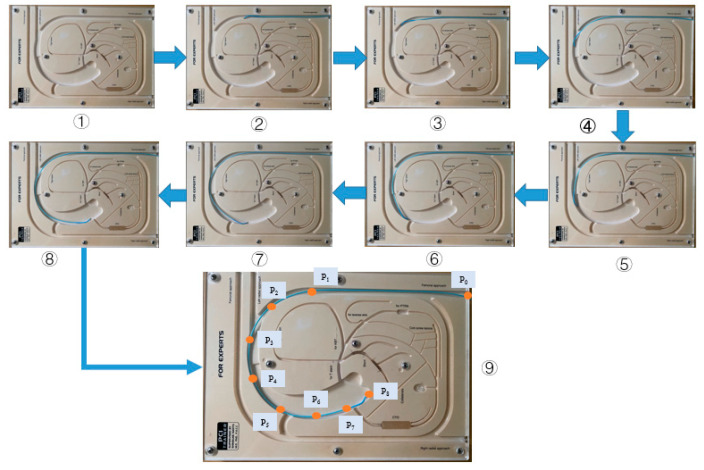
Operational process of the catheter. ①–⑨ are the different positions on the PCI trainer in process.

**Figure 15:**
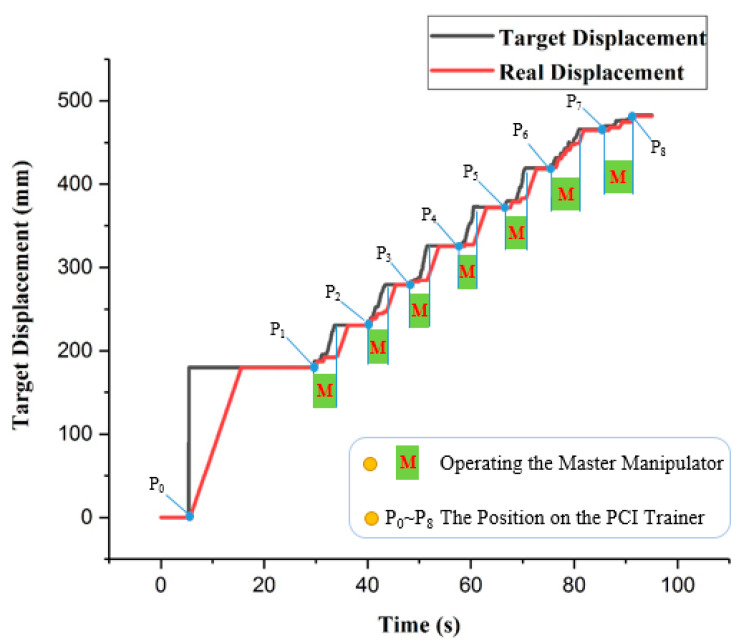
Operational displacements of the master and slave manipulator.

In this section, the evaluation experiments of the ADRC method are presented. The experimental setup is illustrated in Figure 13. The purpose of the experiments is to evaluate the tracking accuracy using the ADRC method. The experimental protocol is as follows: the catheter is driven by the catheter driver module from the initial position $P_{0}$ to the target position $P_{8}$, as shown in Figure 14. In addition, the catheter driver module on the slave manipulator is controlled by the master manipulator in real time. In the experiments, the displacement generated by the master manipulator is the input of the ADRC method, which is the $Dis_{in}$ in Figure 10. Moreover, the working method of the guidewire driver module is the similar to that of the catheter driver module. Therefore, the guidewire driver module is not included in the evaluation experiments. The target and real displacements of the entire operating process are shown in Figure 15. On the percutaneous coronary intervention (PCI) trainer, the characteristic from the initial position $P_{0}$ to the second position $P_{1}$ is a straight line. Therefore, this distance is set up by the serial port assistant, to reduce the operating time and improve efficiency. Meanwhile, in a comparison of the movement characteristics of the two curves in $P_{1}$ to $P_{6}$ and $P_{6}$ to $P_{8}$, the results show that the lower speed of the master manipulator is helpful for the following movement of the catheter driver module in real time.

## 5. Discussion and Conclusions

Operating safety is the most important issue to be considered in the development of the master–slave VISRS. In addition, high tracking accuracy is an efficient method. In this study, the ADRC method is applied in the VISRS. A comparison of the performance of the ADRC method with the incremental PID method shows that the modification time and accuracy of the ADRC method are better. The salient feature is that there is no overshoot when using the ADRC method, which can provide better security.

Moreover, performance evaluation experiments for the VISRS using the ADRC method are implemented. In the experiments, the PCI trainer and the proposed master manipulator are implemented. The experimental results show that if the master manipulator moves with a lower movement speed, the tracking accuracy of the slave manipulator is much better, making it more conducive to the clinical operation than the traditional method.

The results of the evaluation experiments and working status of the master and slave manipulators are shown in Figure 15. Because of the limited distance of the master manipulator, it is necessary to move several times to reach the target position. The maximal error of the tracking is 0.87 mm, and the cumulative error for the entire operation is 1.58 mm. The results show that the ADRC method performs well in completing the operation. Moreover, tactile perception is also necessary and important in master–slave VISRS. Based on the Level of Clinical Realism (LoCR) scale [49,50], the current approach outlined in this study is in the catagory LoCR 1: Training tasks with rigid phantoms. In addition, we will use the ADRC method to complete more complicated surgical tasks in the more realistic environments. In future work, evaluation experiments will be implemented in the EVE model using the ADRC method to realize more complicated operations. In addition, the force feedback device will be evaluated and implemented.
